# Context, complexity and process in the implementation of evidence-based innovation: a realist informed review

**DOI:** 10.1186/s12913-020-4935-y

**Published:** 2020-02-03

**Authors:** K. D. Dryden-Palmer, C. S. Parshuram, W. B. Berta

**Affiliations:** 1 Institute of Health Policy, Management and Evaluation, University of Toronto, Toronto, Canada; 2 Critical Care Program, The Hospital for Sick Children, 555 University Avenue, Toronto, M5G 1X8 Canada; 3 Child Health Evaluative Sciences, Research Institute, The Hospital for Sick Children, Toronto, Canada

**Keywords:** Implementation, knowledge translation, evidence utilization, context, complexity, health care, innovation

## Abstract

**Background:**

This review of scholarly work in health care knowledge translation advances understanding of implementation components that support the complete and timely integration of new knowledge. We adopt a realist approach to investigate what is known from the current literature about the impact of, and the potential relationships between, context, complexity and implementation process.

**Methods:**

Informed by two distinct pathways, knowledge utilization and knowledge translation, we utilize Rogers’ Diffusion of Innovations theory (DOI) and Harvey and Kitson’s integrated- Promoting Action on Research Implementation in Health Service framework (PARIHS) to ground this review. Articles from 5 databases; Medline, Scopus, PsycInfo, Web of Science, and Google Scholar and a search of authors were retrieved. Themes and patterns related to these implementation components were extracted. Literature was selected for inclusion by consensus. Data extraction was iterative and was moderated by the authors.

**Results:**

A total of 67 articles were included in the review. Context was a central component to implementation. It was not clear how and to what extent context impacted implementation. Complexity was found to be a characteristic of context, implementation process, innovations and a product of the relationship between these three elements. Social processes in particular were reported as influential however; descriptions of how these social process impact were limited. Multiple theoretical and operational models were found to ground implementation processes. We offer an emerging conceptual model to illustrate the key discoveries.

**Conclusions:**

The review findings indicate there are dynamic relationship between context, complexity and implementation process for enhancing uptake of evidence-based knowledge in hospital settings. These are represented in a conceptual model. Limited empiric evidence was found to explain the nature of the relationships.

## Background

Clinicians and health services researchers are highly proficient at generating new evidence to inform health care and are notably less effective at moving that new knowledge into practice, thus missing the potential of that research to enhance clinical practice [1, 2]. Persistent calls from implementation scientists and practitioners seek research that contributes to a better understanding of implementation approaches; specifically, understanding why some efforts to implement evidence-based innovation uptake succeed while others fail [2–4]. This review responds to these calls.

There is a large volume and scope of literature describing and evaluating knowledge translation and implementation. Multiple terms have been identified to describe the process of moving research-based knowledge into practice [2, 5]. For our review we selected the term knowledge translation to best describe implementation activities in the acute hospital setting. Knowledge translation is the exchange, synthesis and application of evidenced-based knowledge within complex systems [6, 7]. In order for knowledge translation to occur knowledge users must be exposed to the new knowledge (typically in the form of an innovation) and an intentional mechanism to move that knowledge into practice must be activated. That mechanism is comprised of processes by which the evidenced-based knowledge is intentionally integrated into practice [8]. Hospital settings are often challenging for operationalizing these processes and despite the clear benefits afforded by that new evidence-based knowledge evidence alone is insufficient to catalyze behavior change in health care providers [4, 9–11]. In this review we look closely at specific elements of implementation in hospital settings and explore context, complexity and process, as they related to acute health care implementation. We seek to discover what is known from the literature about the role of context, complexity and process and understand how each might influence the others in the implementation of evidence-based clinical interventions in acute health care settings.

The decision to focus on these three concepts in knowledge translation reflects that each has been acknowledged as influential in health care implementation and each have practical implications for implementers and knowledge users alike (clinicians, administrators, educators) [8].

Context is recognized as an influential component in the uptake of new evidence [12–15]. It has been endorsed as a central construct in conceptual frameworks for implementation such as the PARIHS framework [3]. Context includes the environment or setting in which the proposed innovation is implemented and characteristics associated with that practice setting [1, 16]. There is no fully consolidated understanding of how contextual modifies or impacts implementation. There have been many calls for rigorous methods to identify how implementation approaches could be applied with sensitivity to differing contextual elements [17, 18]. We respond to these calls and highlight context as a key component in this review.

Complexity is acknowledged as a component of the evidence and the resulting innovations (innovation complexity), the implementation processes for the integration of innovations (implementation complexity) as well as a characteristic of the health care environment (context complexity). Innovation complexity occurs when the desire practice change involves multiple steps, multiple stakeholders, and the need for actions across group and teams in an organization [19]. It can also reflect the degree of difficulty in understanding and operationalizing the desired knowledge user behaviours [20].

Implementation complexity reflects the processes and interventions initiated to operationalize the new knowledge into practice. Multiple knowledge users, the presence of tightly held existing practices, multidimensional or interdependent user relationships and diverse settings within an organization contribute to this complexity [14, 21, 22].

Complexity arising from the health care setting in which the implementation activities are situated is ‘context complexity’. Health care organizations are proposed to be amongst the most complex of environments for knowledge translation [19]. Hierarchical reporting structures, multiple local practice cultures, disciplinary cultures and norms, external influences (political, legislative) all contribute to this complexity [23, 24]. These complexities can render implementation outcomes vulnerable to modification, erosion, incomplete uptake and a return to pre- implementation behaviours [25].

The final concept of focus in this review is process. Process describe the way(s) evidence is introduced and facilitated to be taken up in practice [26]. In health care innovation these process are the implementation activities that express the attitudes, beliefs and ways of working of individuals and groups of knowledge users [1]. Process is both the formal and informal mechanisms used to support the application of the innovation and the resulting practice changes [27]. Processes are active across all phases of implementation; pre-implementation, early, active, late and post implementation actions and serve to facilitate and consolidate new provider behaviors [28].

Knowledge translation theories grounding this review theories were drawn from two similar yet distinct conceptualizations of how evidence is moved into practice; 1] research utilization and 2] knowledge translation [29]. Research utilization is informed by Roger’s Diffusion of Innovations theory and describes knowledge generation and translation as relatively context-free [4]. In this pathway evidence-based knowledge is generated independent of the users and the intended setting for use. Movement is unidirectional and predominately linear and proceeds in a stepwise fashion. These steps include awareness of the new evidence, followed by learning, trialing and deciding to adopt, reject or modify that evidence and finally reinforcing the decision. The implementation in this model is thought to be impacted by the qualities of the innovation itself (relative advantage, compatibility, complexity, trialability, observability, flexibility), factors associated with the environment where the introduction of the innovation is taking place (fit to existing system, readiness for change), and the influences of the context and social system in which the change is taking place (resources linkages, central and externalized networks, relationships, champions, opinion leaders). This theory acknowledges the complexity of implementation of evidenced-based innovation and that context and process influence diffusion in a logical sequence [4, 30]. This pathway has been widely studied in nursing and medicine [3, 31].

The second knowledge translation pathway is informed by Kitson’s PARIHS framework that emphasizes an exchange and synthesis of knowledge leading to innovation adoption [3, 8]. This framework highlights the main constructs of evidence, context, facilitation and recipients [3]. In this framework evidence is broadly inclusive of both research generated and experiential knowledge. Movement of evidence into practice is described as non-linear and is influenced by a multitude of factors that interact in sometimes unpredictable ways [32]. These factors exist within the ‘context’ construct whereas ‘facilitation’ captures the active processes that integrate and connect the remaining three. PARIHS acknowledges that evidence-based innovations are fitted with intentional consideration of the context for application and highlights the role of evidence users and their role in implementation. This knowledge translation pathway suggests that the evidence-informed innovation and the context of application co-evolve over time.

## Methods

We used a modified realist-informed review methodology to investigate what is known from the literature about the impact of, and the potential relationship between context, complexity and processes [33]. Realist review is an explanatory approach designed to explore the ‘how and why’ of a complex phenomenon, why things work - or don’t work in a particular context or setting. We applied a realist lens in order to explore a broad scope of evidence such that we might surface emerging trends, gaps and expose both known and unknown impacts of these elements on hospital knowledge translation and implementation. A realist perspective is suited to answering questions of how, for whom and under what circumstances do context, complexity and process impact health care knowledge translation and is a suitable approach for this broad inquiry. The realist-informed perspective is aligned with our interest in the interrelatedness of the concepts and creates opportunities to uncover unanticipated consequences and relationships. We synthesised the findings into a relational representation of the main constructs however did not extend our review to included theory testing.

We applied a modified five-step approach; *planning* that describes the search scope and question refinement, *searching* that identifies the search methods, combined the *mapping* and *appraisal* steps to report on findings, and lastly a *synthesis* for discussion that expands on the implications, value and limitations of the review [33].

*1] Planning:* First we refined the scope for the review to focus on implementation research relevant to the three concepts and to the acute care setting [25, 33]. An exploratory search grounded in the two theoretical pathways was executed in MEDLINE database, results were reviewed and search terms refined. Table 1 contains the search terms.

**Table 1:** Search Terms

Search	Item	Search Terms
Exploratory	Theoretical Pathways	knowledge translation, research utilization
Refined search	Knowledge Translation	knowledge translation, knowledge transfer, knowledge exchange, knowledge dissemination, knowledge application, knowledge cycle, delivery. Knowledge management
	Research Utilization	research; utilization, transfer, translational; science, medicine, implementation science
	Complexity	complex, complexity, complex interventions
	Knowledge Translation Interventions	implementation, barriers, facilitators, guidelines, interventions, education, continuing education, coach, champions, change leader knowledge broker, audit and feedback
	Context	acute care, hospital
	Process	change, adoption, innovation adoption, program change, research-practice gap, behavioral change, reform
	Authors	Straus, S., Greenhalgh, T., Graham, I., Grimshaw, J., Berta, W., Kitson, A., Estabrooks, C., Logan, J., Rogers, E., Pettigrew, M., Pawson, R., Grol, R., Fineout-Overholt, Raycroft-Malone, J.
	Additional terms in final search	knowledge use, policies, spread, quality improvement, best practice, organization, system, integrate, (removed learning)

2] Searching: Searches of 5 electronic of databases and online sources including: MEDLINE (CINAHL), Scopus, PsycInfo, Web of Science, and Google Scholar were carried out with an experienced academic health services librarian. Electronic searches were limited to English language and full text availability. An author search was conducted targeting specific scholars in the knowledge translation and implementation fields as identified by the senior reviewer and through discussion with local knowledge translation scientists. Further searches of the Joanna Briggs and McMaster University knowledge translation web sites were carried out. The searches were carried out in 2015 and refreshed in 2017.

Electronic search results were loaded into End Note 7.0 for cataloging. Preliminary screening of returned articles for duplicate and non-English documents was conducted. The remaining citations were compiled in a screening document, for inspection by the three reviewers. Articles were screened for inclusion of the concepts such that one of 4 criteria were met; 1] the article addresses context, 2] the innovation or the setting is complex, 3] the article addresses complexity, and 4] there are process measures or discussion of process. Outcomes of implementations were not in scope for this review. Studies situated in the developed world were selected to best represent the context of contemporary acute health care settings and publications from 1997 onwards due to the sharp increase in publications in the field at that time. Both theoretical and empirical work was included. We excluded educational program reports, individual level learning reports, simple innovations information technology projects as well as any remaining duplicates, books, book chapters and conference proceedings. Eligibility criteria were applied to the remaining documents (Table 2). Additional file 1 provides the complete search strategies by database.

**Table 2:** Inclusion and Exclusion Criteria

	Inclusion criteria	Exclusion criteria
Context /setting:	Health care focus and acute care setting	No health care focus, non-acute care setting
Level of measure:	Minimum one organization measure	No discussion/measures/outcomes at organizational level
Addresses a knowledge translation innovation:	Yes	No
Has evidence of complexity:	Multiple stakeholders Involves actions of multiple people/teams Chains or steps in a process Non-liner processes Embedded in social systems Prone to modification or change	Does not meet complexity criteria
Location:	Developed world Stable health care system-comparable to Canadian system acute care	Developing world, health care context without stable health care infrastructure
Intervention types:	Multi, program or complex innovation(s)	Single or simple innovations/or technology product
Study/publication types:	Empirical, theoretical, expert opinion, reviews	Conference proceedings, books and book chapters, unpublished work
Accessibility:	English language, retrievable	Non-English, non-retrievable
Addresses factors of interest:	Context, complexity and process	Does not address context, complexity and process

3] Mapping: Each abstract was screened independently by 2 of the 3 reviewers. Articles without abstracts were reviewed as full text.

Consistent with recommend realist review methodology the quality of the studies was attended to in terms of ‘fit’ to the review purpose and no explicit quality ranking was pursued. Realist reviews focus on relevance of included articles to establish usefulness in the context of the specific question at hand rather than the application of formal study design quality criteria [25]. A sample of ten articles (approx15%) were screened and compared by all three reviewers to check internal constancy within the group and to clarify interpretation of fit and application of the inclusion and exclusion criteria.

Data extraction of eligible studies proceeded in two phases. The first review abstracted the articles for study type (empirical, theoretical, opinion or review), method (if applicable), main focus and findings. The second phase of abstraction involved a re-reading of each article for findings that addressed context, complexity and process. Findings were then grouped into themes for further analysis. Analysis was constant and comparative in phase two such that emerging findings in the new data initiated a further analysis for similar and disparate data in the previously reviewed items.

## Results

**Fig. 1:**
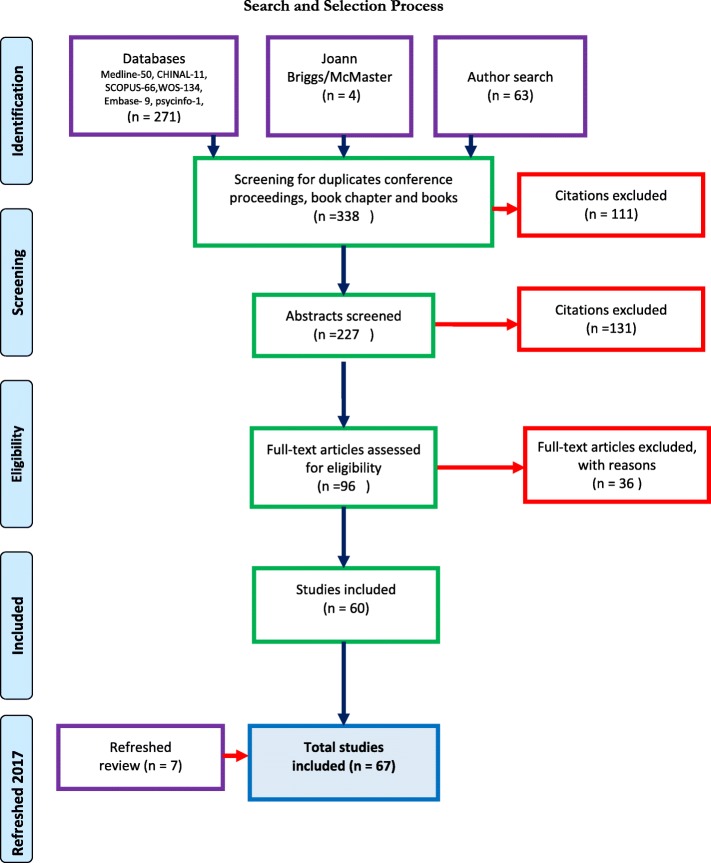
Search and Section Process: This diagram outlines the process of article review and selection for inclusion in analysis

The initial search referred a total of 338 electronic references for inclusion and 111 citations were removed after sorting to remove duplicates, conference proceedings, book chapter and books, The remaining 227 references were prepared for abstract screening and a total of 96 citations were included for full text screening. After full text review was completed 36 references were excluded and 60 articles remained for appraisal. The subsequent refreshing of the searches in 2017 added 7 articles for a total of 67. Figure 1 shows the flow of the search through screening and article selection.

3] Appraisal: There was wide representation from multiple health care disciplines and health services with a total of 42 journal titles represented in the sample. Clinical and professional journals (medicine-14, nursing-14) were the source of the majority of citations (*n* = 28) followed by health services research journals (*n* = 15). Implementation science journals contributed 10 citations and quality improvement (*n* = 4), policy, education and leadership with 3 citations each. The articles returned were evenly distributed between study types. There was a heterogeneity of designs noted in the sample and implementation outcomes were diverse and inconstantly reported.

A total of 21 evidence-generating studies included 7 case studies. Of the empiric citations representation was evenly distributed between quantitative [7], qualitative [7] and mixed/multi methods [7] approaches. Nine theoretical papers were returned and a further ten focused on model building or testing. Citations included 16 reviews and 11 expert opinion papers. Table 3 provides a listing of authors, titles, journal and domain of the included citations.

**Table 3:** Articles Table (*n* = 67)

Author	Year	Title	Type	Journal	Domain
Armstrong R. et.al.	2013	Knowledge translation strategies to improve the use of evidence in public health decision making in local government: Intervention design and implementation plan.	Review	Implementation Science Oct 9; 8:121	Implementation
Barnsley J. et al.	1998	Integrating learning into integrated delivery systems	Theoretical	Health Care Management Review Winter; 23 (1):18–28	Health Services Research
sBerta WB. et al.	2004	Factors that impact the transfer and retention of best practices for reducing error in hospitals.	Expert opinion/ Persepctive	Health Care Management Review Apr-Jun; 29 (2):90–7	Leadership
Berta W. et al.	2015	Why (we think) facilitation works: insights from organizational learning theory.	Expert opinion/ Persepctive	Implementation Science Oct 6, 10:141	Implementation
Carayon P.	2010	Human factors in patient safety as an innovation.	Model building-Testing	Applied Ergonomics 41 (5): 657–65	Health Services Research
Chor KH. et al.	2015	Measures for Predictors of Innovation Adoption.	Model building-Testing	Administration and Policy in Mental health Sep; 42 (5):545–73	Health Policy
Cochrane LJ. et al.	2007	Gaps between knowing and doing: understanding and assessing the barriers to optimal health care.	Review	Journal of Continuing Education in the Health Professions Spring; 27 (2): 94–102	Education
Cummings GG et al.	2007	Influence of organizational characteristics and context on research utilization.	Model building-Testing	Nursing research Jul-Aug; 56 (4 Suppl):S24–39	Nursing
Davis D. et al	2003	The case for knowledge translation: shortening the journey from evidence to effect.	Expert opinion/ Persepctive	BMJ Clinical research Jul 5; 327 (7405):33–5.	Medicine
Denis JL. et al.	2002	Explaining diffusion patterns for complex health care innovations.	Case Study Mixed or multi-method	Health Care Management Review Summer; 27 (3):60–73	Leadership
Dijkstra R. et al.	2006	The relationship between organisational characteristics and the effects of clinical guidelines on medical performance in hospitals, a meta-analysis.	Review	BMC Health Services Research Apr 28; 6:53	Health Services Research
Dopson S.	2005	The diffusion of medical innovations: Can figurational sociology contribute?	Theoretical	Organization Studies 26 (8):1125–44	Implementation
Eccles M. et al.	2005	Changing the behavior of healthcare professionals: the use of theory in promoting the uptake of research findings	Model building-Testing	Journal of Clinical Epidemiology 2005;58 (2):107–12	Health Services Research
Eldh AC. et al.	2013	Translating and testing the Alberta context tool for use among nurses in Swedish elder care.	Empirical Mixed or multi-method	BMC Health Services Research 13:68	Health Services Research
Estabrooks CA. et al.	2003	Measuring knowledge utilization in health care.	Review	Int J Policy Eval Manage. 2003; 11 (3):3–36	Health Policy
Estabrooks CA.	2006	A guide to knowledge translation theory.	Theoretical	Journal of Continuing Education in the Health Professions 26 (1):25–36	Education
Estabrooks CA.	2007	Prologue: a program of research in knowledge translation.	Expert Opinion/Persepctive	Nursing Research 56 (4 Suppl):S4–6	Nursing
Ferlie E. et al.	1999	Some limits to evidence-based medicine: A case study from elective orthopaedics.	Case Study Qualitative	Quality in Health Care 8 (2): 99–107	Quality Improvement
Fineout-Overholt E. et al.	2004	Strategies for advancing evidence-based practice in clinical settings.	Case study Quantitative	The Journal of the New York State Nurses’ Association 7 (1):51–3	Nursing
Fineout-Overholt E. et al.	2010	Teaching EBP: strategies for achieving sustainable organizational change toward evidence-based practice.	Expert opinion/ Persepctive	Worldviews on Evidence-based Nursing 35 (2):28–32	Nursing
Foy R. et al.	2002	Attributes of clinical recommendations that influence change in practice following audit and feedback	Empirical Quantitative	Journal of Clinical Epidemiology 55 (7):717–22	Health Services Research
Foy R. et al.	2005	Theory-based identification of barriers to quality improvement: induced abortion care.	Empirical Mixed or multi-method	International Journal for Quality in Health Care 17 (2):147–55	Quality Improvement
Franx G. et al.	2014	Organizational change to transfer knowledge and improve quality and outcomes of care for patients with severe mental illness: a systematic overview of reviews.	Review	Canadian Journal of Psychiatry 53 (5):294–305	Medicine
Gagnon MP. et al.	2011	Measuring organizational readiness for knowledge translation in chronic care.	Review	Implementation Science Jul 13; 6:72	Implementation
Graham ID. & Logan J.	2004	Innovations in knowledge transfer and continuity of care.	Expert opinion/ Persepctive	The Canadian Journal of Nursing Research 36 (2):89–103	Nursing
Graham ID et al.	2006	Lost in knowledge translation: time for a map?	Theoretical	Journal of Continuing Education in the Health Professions 26 (1):13–24	Education
Graham ID. et al.	2007	Some Theoretical Underpinnings of Knowledge Translation.	Theoretical	Academic Emergency Medicine 14 (11):936–41	Medicine
Greenhalgh T. et al.	2004	Diffusion of innovations in service organizations: systematic review and recommendations	Review	Milbank Quarterly 82 (4):581–629	
Greenhalgh T. et al.	2005	Storylines of research in diffusion of innovation: a meta-narrative approach to systematic review.	Review	Social Science & Medicine 61 (2):417–30	Medicine
Grimshaw JM. et al.	2001	Changing provider behavior: an overview of systematic reviews of interventions.	Review	Medical Care 39 (8 Suppl 2):Ii2–45	Medicine
Grol R.	2001	Successes and failures in the implementation of evidence-based guidelines for clinical practice.	Empirical Quantitiative	Medical Care 39 (8 Suppl 2):II46–54	Medicine
Grol R. et al.	2003	From best evidence to best practice: effective implementation of change in patients’ care.	Expert opinion/ Persepctive	Lancet 362 (9391):1225–30	Medicine
Grol R. et al.	2004	What drives change? Barriers to and incentives for achieving evidence-based practice.	Theoretical	Medical Journal of Australia 180 (6 Suppl):S57–60	Medicine
Harrison MB. et al.	2013	Guideline adaptation and implementation planning: A prospective observational study.	Empirical Mixed or multi-method	Implementation Science 2013;8 (1)	Implementation
Harting J. et al.	2005	Implementation of an innovative health service - A “real-world” diffusion study	Case study Qualitative	American Journal of Preventive Medicine 29 (2):113–9	Medicine
Harvey G. et al.	2016	PARIHS revisited: from heuristic to integrated framework for the successful implementation of knowledge into practice.	Model 5building-testing	Implementation Science Mar 10;11:33	Implementation
Kastner M. Straus SE.	2012	Application of the Knowledge-to-Action and Medical Research Council frameworks in the development of an osteoporosis clinical decision support tool.	Model building-testing	Journal of Clinical Epidemiology 65 (11):1163–70	Health Services Research
Kerner JF.	2008	What we see depends on where we stand.	Expert opinion/ Persepctive	Journal of Public Health Management and Practice. 14 (2):193–8	Health Services Research
Kerner JF. Hall K.L	2009	Research Dissemination and Diffusion:Translation within Science and Society	Theoretical	Research on Social Work Practice 19 (5):519–30	Health Services Research
Kitson A. et al.	1998	Enabling the implementation of evidence based practice: a conceptual framework.	Model building- testing	Quality in Health Care 7 (3):149–58	Quality Improvement
Kitson A. & Straus SE.	2010	The knowledge-to-action cycle: identifying the gaps	Expert opinion/ Persepctive	CMAJ Canadian Medical Association Journal 182 (2):E73–7	Medicine
Latimer MA. et al.	2010	Individual nurse and organizational context considerations for better knowledge use in pain care.	Model building-testing	Journal of Pediatric Nursing 25 (4):274–81	Nursing
Lekan D. et al	2010	The Connected Learning Model for disseminating evidence-based care practices in clinical settings.	Model building-testing	Nurse Education in Practice 10 (4):243–8	Nursing
Lemieux-Charles L. et al.	2002	Building interorganizational knowledge for evidence-based health system change.	Case study Mixed or multi-method	Health Care Management Review 27 (3):48–59	Health Services Research
Matthew-Maich N. et al.	2013	Supporting the uptake of nursing guidelines: What you really need to know to move nursing guidelines into practice	Empirical Qualitative	Worldviews on Evidence-based Nursing 10 (2):104–15	Nursing
McCormack B. et al.	2002	Getting evidence into practice: the meaning of ‘context’.	Theoretical	Journal of Advanced Nursing 38 (1):94–104	Nursing
McCormack B. et al.	2013	A realist review of interventions and strategies to promote evidence-informed healthcare: a focus on change agency.	Review	Implementation Scienc 8:107	Implementation
McKibbon KA. et al.	2006	A cross-sectional study of the number and frequency of terms used to refer to knowledge translation in a body of health literature in 2006: a Tower of Babel?	Empirical Quantitiative	Implementation Science 5:16	Implementation
Novotna G. et al.	2012	Institutionalization of evidence-informed practices in healthcare settings	Expert opinion/ Persepctive	Implementation Science 7:112	Implementation
Oborn E.	2012	Facilitating implementation of the translational research pipeline in neurological rehabilitation.	Review	Current Opinion in Neurology 25 (6):676–81	Medicine
Petticrew M. et al.	2013	Complex interventions and their implications for systematic reviews: a pragmatic approach	Review	Journal of Clinical Epidemiology 66 (11):1209–14	Health Services Research
Rangachari P.	2008	The strategic management of organizational knowledge exchange related to hospital quality measurement and reporting	Empirical Qualitative	Quality Management in Health care 17 (3):252–69	Quality Improvement
Retsas A.	2000	Barriers to using research evidence in nursing practice.	Empirical Quantitative	Journal of Advanced Nursing 31 (3):599–606	Nursing
Sanson-Fisher RW.	2004	Diffusion of innovation theory for clinical change.	Theoretical	Medical Journal of Australia 180 (6 SUPPL.):S55-S6	Medicine
Scott SD. et al.	2008	Factors influencing the adoption of an innovation: An examination of the uptake of the Canadian Heart Health Kit	Case Study Qualitative	Implementation Science 3 (1)	Implementation
Scott SD. et al.	2011	Optimizing clinical environments for knowledge translation: strategies for nursing leaders.	Empirical Qualitative	Nursing Leadership 24 (3):73–85	Nursing
Scullion PA.	2002	Effective dissemination strategies	Expert opinion/ Persepctive	Nurse researcher 10 (1):65–77	Nursing
Sibbald SL. Et al.	2013	Knowledge flow and exchange in interdisciplinary primary health care teams (PHCTS): An exploratory study	Empirical Mixed or multi-method	Journal of the Medical Library Association 101 (2):128–37	Medicine
Snyder-Halpern R.	1998	Measuring organizational readiness for nursing research programs.	Model building-testing	Western Journal of Nursing Research 20 (2):223–37	Nursing
Soper B. et al.	2013	CLAHRCs in practice: combined knowledge transfer and exchange strategies, cultural change, and experimentation.	Empirical Mixed or multi-method	Journal of Health Services Research & Policy 18 (3 Suppl):53–64	Health Services Research
Straus SE. et al.	2011	Knowledge translation is the use of knowledge in health care decision making.	Review	Journal of Clinical Epidemiology 181 (3–4):165–8	Health Services Research
Wallin L.	2006	Development and validation of a derived measure of research utilization by nurses.	Empirical Quantitative	Nursing Research 55 (3):149–60	Nursing
Ward V. et al.	2009	Developing a framework for transferring knowledge into action: A thematic analysis of the literature.	Review	Journal of Health Services Research and Policy 14 (3):156–64	Health Services Research
Weigel FK. et al.	2014	Diffusion of innovations and the theory of planned behavior in information systems research: A metaanalysis.	Review	Communications of the Association for Information Systems 34 (1):619–3	Leadership
Weiner BJ. et al.	2007	Adoption and implementation of strategies for diabetes management in primary care practices.	Case Study Qualitative	American Journal of Preventive Medicine 33 (1 Suppl):S35–44; quiz S5–9	Medicine
Wensing M. et al.	2010	Developing and selecting interventions for translating knowledge to action.	Review	CMAJ Canadian Medical Association Journal 182 (2):E85–8	Medicine
Yousefi-Nooraie R. et al.	2012	Information seeking for making evidence-informed decisions: a social network analysis on the staff of a public health department in Canada	Empirical Quantitative	BMC Health Services Research 12, 118	Health Services Research

Table 3 **text.**

A total of 42 separate journals composed the articles retrieved. Health services research was most represented with 15 citations. Medical and nursing journals were next with 14 citations in each. Implementation science publications were next with 10 citations followed by 4 citations in quality improvement and 3 each found in policy, education and leadership journals. Empirical studies numbered 21 with the majority being case reviews [7]. Within the empirical group qualitative, quantitative and mixed or multi-method approaches were evenly distributed at 7 each. Nineteen citations were theoretical [9] or model building/evaluating [10]. Reviews made up a further 16 citations with 11 expert opinion papers completing the sample

A number of research traditions and approaches were represented in the search results, for example social network analysis; diffusion of innovation theory, theory of planned behaviours and human factors. There was consensus across the majority of papers that getting evidence into clinical practice is complex and does not follow a prescribed, ‘logical’ path thus creating theoretical and practical changes for implementation planning and evaluation [34–36]. Theoretical opacity, multiplicity of terminology in the domain, multiple variables and processes in practice were cited as specific confounders to the study of health care implementation [26, 37]. We noted a lack of distinction between context, complexity and process in the reviewed articles confirming the often-described pragmatic difficulties in defining and quantifying factors of influence [19]. Distilling or isolating context, complexity and process form other factors in the studies was recognized as somewhat artificial and potentially limiting implementation research [25]. For example, a number of papers concluded that successful knowledge translation is heavily reliant on relationships and social influences, however those very relationships are shaped in part by the contexts in which they occur and the processes in which organizational actors participate [20, 38]. Therefore, to conceptualize processes as discrete from context may potentially distort how we understand the mechanisms of influence at work in implementation. Acknowledging this limitation we proceeded with analysis of each concept individually and the relationship between them.

### Context

Examples of context in the reviewed articles included group culture, the history of the community in which evidence-based innovation is implemented, the nature and scope of existing relationships, social networks, and organizational structure [8]. Context was often described as pre-existing and as either a barrier or facilitator of implementation. Discussion related to context urged implementers to understand and respond to context, including the forces that give the physical, social and political environment its character [15, 39]. To achieve optimum innovation adoption establishing a context that values evidence in guiding practice was recommended [12, 29, 40]. Context was most often conceptualized as a modifiable, although difficult to change [16, 38, 41, 42]. Failure to address context was the most commonly cited consideration in implementations that failed to achieve the desired goals [35, 43].

Modifications to the innovation being introduced as well as modifications in the setting were both noted as helpful implementation interventions. The former achieves contextualization of the innovation and the later alters the context of the practice environment opening an opportunity for change [1, 44]. Both approaches were seen to enhance the compatibility between the innovation and the context for use [45]. Context modifications were most often seen in the empirical studies [43]. Context was also described as a relational component of the organization often expressed as leadership styles that can enable or hinder adoption [46, 47]. Transformation leadership in particular was thought to promote an evidence-positive context and improve change culture.

Culture emerged as an important element of context. Leadership, organizational culture for change and evidence utilization were cited as having both negative and positive impacts on implementation outcomes [46, 48]. For example an organizational culture of uncertainty was described as unfavourable to evidence-based practice. High cultural cohesiveness was negatively associated with guideline adherence [49]. A number of papers suggested that leadership interventions could modify cultural uncertainty related to evidence uptake and promote a more stable context for the introduction of innovations specifically enhancing a supportive social structure, shared values, role clarity, policy development and change motivation [42, 50, 51]. Cultural context was reported to be experienced at the individual level and at the organizational level. Typologies of organizational culture were described in relation to implementation approaches (clan, hierarchical, development and rational) [10, 20]. Interestingly, high cultural cohesiveness in a group was seen as associated both with readiness for evidence-based change and resistance to that change.

Context was also proposed as shaping the evidence and the innovation itself [20]. Existing values, past experience and the knowledge user needs were identified as contextual elements that influenced implementation through acting on the evidence or innovation itself. Individual level strategies to adapt new knowledge this enhancing individual practice ‘fit’ with practice context were noted [49, 52]. Rycroft-Malone et al. (2004) described knowledge translation as shaping the knowledge to fit at all levels, individual, team and organizational [31]. The active engagement of the knowledge user is posited to support stakeholder interest, acceptance of new practices and ownership of the outcomes, provided that organization inhibitors are attended too [10, 53]. Informal connections between individuals and teams that allow for sharing of evidence-based innovations and practices were commonly reported as important although not often evaluated [54]. The action of ‘fitting the evidence’ facilitated a sense of end user ownership of the new practice(s) and normalized the innovation in the environment.

Calls for the tailoring of implementation processes in order to facilitate innovation fit were prevalent, however evidence demonstrating the impact of this tailoring was mixed [1, 44, 45, 52, 55, 56]. Context was described as impacting implementation although little attention was paid to how context interfaces with the implementation processes leading to those outcomes [56–58]. Gagnon et al. (2010) for example, identified that tension to change, innovation fit to context and resource availability interact to play a role in implementation outcomes but did not address how these factors might shape the implementation actions and processes [22].

### Complexity

Complexity emerged as a feature of the context in which implementation occurred, a characteristic of the implementation process itself and an attribute of the innovation being implemented. Complexity was also described as arising from complex questions, complex interventions and complex hypothesized mechanisms of influence [19, 29]. Complexity was broadly acknowledged as a key element in health care knowledge translation although we found a lack of specificity around the role that complexity plays in implementation. Denis et al. (2002) suggested that there is a concerning dearth of research that addresses complexity’s role in implementation and they were critical of perspectives that view complexity as a fixed characteristic of context [29]. They advocated for complexity to be viewed on a continuum across all elements of implementation. While complexity was repeatedly mentioned as a characteristic of the evidence, the innovation and the setting, it was not reported on or evaluated in terms of impact. Calls for increased attention to complexity and the practical impacts of complexity were prevalent in the sample [21, 28, 57, 58].

Implementation complexity was attributed to the non-linear, evolving implementation processes that are unpredictable relative to contextual influences and individual user needs across different settings [32, 57]. Complexity in implementation was generally attributed to the multiple stakeholders (clients, health care providers, decision-makers, and communities) with differing roles and a variety of accountabilities related to the desired evidence-based change(s) [22, 57]. Each stakeholder was impacted by explicit and implicit processes that intersect at individual, team, and organizational levels thus creating a matrix of complexity and the necessity to design implementation processes that address the nuances of different end users.

### Process

Multiple models and approaches to implementation process were noted in the sample. Ward et al. 2009 review of knowledge translation theory identified 28 different models that were explanatory or grounding knowledge translation interventions and implementation process [38]. The resulting lack of conceptual clarity, liberal discipline-specific language, limited theory testing and theory merging has made study of the process difficult [59]. This opacity has limited our ability to discern which processes are most effective and in what contexts.

There were a variety of conceptualizations of, and frameworks for guiding implementation including theoretical, phased, and cyclic models [28, 60–62]. Rogers theory of diffusion is widely acknowledged as the guiding framework in the majority of the intervention articles [4]. Other theoretical and empirical work cited was the Knowledge to Action framework and the PARIHS model [18, 63, 64]. Consolidated typologies of knowledge translation frameworks emerged and included diffusion of innovations (linear), evidence-based medicine model (cyclic), social interactional models and the knowledge utilization process (multi-directional as well as multiple level process [65]. Phased implementation models were suggested as a practical way to minimize and deconstruct the complexity of implementation processes and achieve enhanced uptake however there was insufficient evidence to favour any particular approach [28]. Outcomes were improved where theoretical and pragmatic guiding frameworks and implementation processes were selected with attention to fit with the context [18].

Much of the implementation processes addressed in the articles were socially mediated processes that were frequently qualified as facilitative [64, 66]. Facilitation was described either as a role played by an implementation participant, a product of interpersonal relationships within the organization and as a specific intentional knowledge translation intervention [8]. Innovation adoption was shown to improve with increased facilitation with emphasis on assisting individual users to understand and apply the evidence [30, 67, 68]. Concurring with these observations about the importance of social interaction, Scott et al. (2008) found that knowledge uptake and integration was decreased for practitioners who work alone [35]. Social facilitation was also posited to positively impact organizational capacity for learning and uptake of innovation [67].

Social processes were suggested to be most beneficial to implementation when knowledge resided within individuals rather than other organizational knowledge reservoirs like organizational structure or informational space [69]. The positive impacts of person-to-person relationship were proposed to accumulate overtime impacting the change culture [29]. Facilitative roles described in the papers included; knowledge broker, mentor, facilitator, champion, evidence-based practice leader, and change agent [10, 46]. Detailed descriptions to differentiate these roles from one another were absent and there was little information on how to operationalize social facilitation for implementation [58]. Social facilitation roles were seen to provide expert knowledge related to the innovation and assist with integrating new practice behaviors through point of use coaching and peer support [46, 70]. Social processes were described as a means to breaking down specific implementation barriers bridging the gap between knowing and knowing-how [71]. Lack of awareness of the evidence, lack of motivation and under-utilized external drivers of change are examples of implementation barriers described in the studies that social processes have positively impacted [55].

## Discussion

4] Synthesis: Context emerged as the central factor in implementation having the broadest impact affecting the evidenced-based innovation, the implementation processes and the implementers/knowledge users. A breadth of observations described situations where context was an active modifier in implementation. For example, a “top down” organizational culture was described as inhibiting uptake in nurses, however the same paper described “top down” support as necessary to launch and sustain implementation initiatives and therefore essential for uptake [72]. These findings suggest that leadership culture can be facilitative and inhibitive. This leads to questions of whether other components of context can be both positively and negatively influencing implementation, suggesting a continuum of action rather than dichotomous good and bad effects.

Context was influenced and potentially altered by the introduction of new evidenced-based innovations and the associated implementation processes. The nature of the relationship between context and implementation processes and by what mechanisms does the evidence modify the context remained unclear. Acknowledging that innovations are modified at the point of care to achieve fit with the immediate patient and provider needs leads one to reflect on the impact that context may have on innovation fidelity. Learning more about how knowledge users weigh and manipulate new information can expose and potentially predict the impact of context. The component elements of context that were highlighted as amenable to modification were not discreetly described. Some author’s call for well-designed prospective studies to better understand context so as to guide implementers to the desirable modifications and potential consequences of intentionally fitting the context to the knowledge being introduced [20, 55]. Theoretical and practical limitations on our understanding of context remain however we can assert that context is more than a backdrop for knowledge translation. Context is a dynamic and active element that responds to and is impacted by both the new knowledge and the implementation process.

Complexity is both a static characteristic of all three elements and arises from the relationship between them. Complexity is an accepted steady state in hospital settings. The mechanism by which complexity influences implementation was not overtly explored. Reducing complexity is thought to be beneficial however how this can be accomplished and to what extent complexity is motivational or inhibitory is not yet defined. Recommendations to plan for and minimize complexity range across enhancing communication, providing tools (decision aids, checklists) and phasing/staging implementation.

Implementation processes were grounded in a wide range of theoretical models and frameworks [39, 73]. Social processes dominated the empirical studies and program reports, specifically facilitative roles like coaching models or train the trainer models. There was general agreement that lack of theoretical clarity and robust description of socially mediated process has limited the evaluation of these processes. Descriptions of how social connections enhanced implementation were not common nor are the operation details of the more effective models. This results in continued confusion over what processes are most effective and in what circumstances.

None of the studies in this review directly addressed or accounted for the relationship between the three elements at the focus of this review. Understandings of how process may modify the context, how context might modify process, or how they are interrelated, how processes impact and are impacted by complexity, and in what ways process ultimately shapes implementation outcomes is incomplete. Continued theory refinement inclusive of the underexplored relationships between context, complexity and process is needed.

**Fig. 2:**
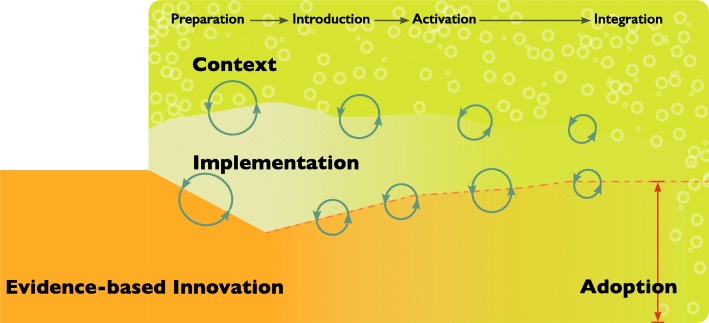
Tunnel model of Implementation Context, Complexity and Process: The figure depicts an evidence-based innovation as it is introduced and moves into an acute care system. As the innovation moves into the system it is exposed to the context of that system. The innovation has intended and unintended impacts on the context of that system as a result of that exposure. The innovation is also impacted by that system as implementers and knowledge users seek to refine and ‘fit’ it to the practice reality. Complexity is represented in the textured background of the context as it is a characteristic of that context. Complexity is also generated in the relationship between the innovation, implementation process and the context. The circular arrows indicate this evolving complexity at the point of interaction between the evidence-based innovation and implementation process and the implementation process and the context. Implementation processes are situated within the context of the system. Some implementation processes refine the context of the system for improved innovation adoption while others are shaped by the existing context. As the innovation is moved through the phases of preparation, introduction, activation and integration towards adoption, intentional and organic implementation process are initiated shaping the context, the implementation processes and the evidence-based innovation. As the innovation moves further towards adoption the reciprocal relationship between context and process and process and innovation alter the shape and fit of the innovation in the new space as it is taken up. The processes fade as adoption is approached and implementation processes are less active. As adoption is achieved the innovation is transformed into an element of the system and becomes part of the context fading as it is normalized

We suggest an emerging conceptual model that reflects the relationship between context, complexity and process that occur with the introduction of evidence-based innovation into practice. Figure 2 illustrates this developing model of the relationships between the new knowledge, the context for implementation and the implementation process on the background of complexity.

As an evidence-based innovation is moved into the system it is exposed to the context of that system. The innovation is designed to impact that system through the associated practice changes. The innovation is also impacted by that system as implementers and knowledge users seek to refine and fit the innovation to their practice reality.

Complexity is both a static characteristic of the innovation, the implementation process and the context and emerges as a component of the dynamic relationship between the innovation, context and implementation process. The circular patterns in the context zone indicate the static complexity in implementation and the circular arrows represent the evolving complexity resulting from the relationship between the elements as the innovation moves towards adoption.

As the innovation moves into the system, intentional and organic implementation processes are initiated. Implementation processes are situated in the context of the system and some implementation process are designed to refine the context of that system for improved innovation uptake, for example nurturing a change culture. Likewise, implementation processes are responsive to the existing context (for example top down leadership context that can expedite access to resources to support new practices). Processes might then be intentionally or organically altered in response to relationships with the context elements.

As the innovation progresses through the implementation stages of preparation, introduction, activation, integration and adoption, the reciprocal relationships between context and process and process and innovation alter the shape and fit of the innovation in the new space as it is taken up. As adoption is achieved the innovation is transformed into an element of the system and becomes a normalized part of the context.

This review reveals that there are opportunities to explore the interface between the individual components of knowledge translation. The impacts of context, process, complexity and the evidence have been studied in isolation of one another, or in simplified dyadic combinations. Continued theory refinement inclusive of the underexplored relationships between context, complexity and process is needed. Research to understand the inter-relationships amongst these elements is needed to expose the relational complexities and interdependencies. In this way the existing (and perhaps artificial) divisions between knowledge translation elements and levels of influence (individual, team, organization, system and innovation) can be bridged.

The studies reporting on implementation outcome measures were few and the measure themselves were heterogeneous suggesting that analysis of construct and factor impacts on implementation outcomes is not currently feasible. Future work that is closely aligned with established conceptual frameworks and consistently accepted outcomes would enhance opportunities for evaluating the effects of implementation components.

The majority citations addressed the components of implementation in terms of barriers and facilitators. This binary approach may potentially be limiting to the how the discrete components are viewed and how their directions of influence are perceived [74]. In this review components that are facilitative in one context may well be a hindrance in another therefore they might be better conceptualized as on a continuum of influence. Future research should resist referencing barriers and facilitators and embrace a non-direction lens to capture all the potential impacts of these factors.

In this review the specific focus on acute care settings, the lack of formal quality appraisal of the included studies and broad scope of study types and the timeline of the review are potential limitations. There scope of include studies was wide as we had intended and there was equal representation between original research papers and theoretical papers achieving a broad perspective. Inclusion of all types of evidence is in keeping with a realist approach however applicability of these broad findings to other settings is difficult to evaluate. In the evidence generating papers there was a variation in the levels of evidence and detail provided such that conclusions related to specific implementation approaches cannot be made. Theoretical papers did not specifically describe nor address the relationships between each element of the review and often the descriptions of the presumed mechanisms of influence on and between the main concepts were absent. Completing the review at the synthesis stage does not allow for evaluation or exploration the proposed model. As a result, theoretical conclusions are generalized.

The review was primary focused on context, complexity and process did not extend to explicitly include the consequences of these elements in implementation outcomes. Although adoption is the natural outcome in this domain we did not look to specify nor explore adoption as a discreet construct.

The review was undertaken originally in 2016 and updated on 2017 and interest in knowledge translation has accelerated. This review reflects the evolution the field at that time and provides important insights in underexplored areas of current focus in implementation science. This study will further solidify the platform for ongoing work in this domain. The Rameses Publication standards for Realist Synthesis was used to guide this report [75].

## Conclusion

This review explored context, complexity and implementation processes in relation to the implementation of evidenced-based innovations. Understanding the influences of these central concepts and their influence is essential to the design of efficient and effective implementation approaches. This review revealed an incomplete understanding about the interface between these three components and described areas for further inquiry. An emerging conceptual model was offered that could help to ground implementation research and practices and inform the realization of the full potential of evidence-based innovation.

## Supplementary information

**Additional file 1.** Search Strategies by database.

## Data Availability

Data sharing is not applicable to this article as no data sets were created or utilized in the review. All citations include in analysis are documented in the references and listed in the citation table.

## Abbreviations

DOI: Diffusion of Innovations theory
PARIHS: Promoting Action on Research Implementation in Health Service framework
