# Supplementary material for: Context, complexity and process in the implementation of evidence-based innovation: a realist informed review
Source: BMC Health Serv Res. 2020 Feb 3;20:81. doi: 10.1186/s12913-020-4935-y (PMC6998254; doi:10.1186/s12913-020-4935-y)
Supplement: Supplementary file 1 — Additional file 1. Search Strategies by database. [file 12913_2020_4935_MOESM1_ESM.docx]

Additional File 1

Search Strategies by Data Base

**Search Strategies**

Executed June 2014- Feb 2015, Revised June 2017

| Medline/  Cinhal | (MH "Knowledge Management"), TX (knowledge N3 (translat* OR exchang* OR transfer* OR diffusion OR disseminat*)), organization* OR organisation*), (MH "Diffusion of Innovation"), (innovat* N3 diffus*), MH "Health Care Delivery, Integrated") OR (MH "Health Care Delivery") OR (MH "Health Care Reform") |
| --- | --- |
| Embase | Knowledge management (knowledge adj3 (translat* or exchang* or transfer* or diffusion or disseminat*)).tw. , complex*.tw. (1190526), organization, (organization* or organisation*).tw., mass communication, (innovat* adj3 diffus*).tw., health care delivery/ or integrated health care system/ (133185) |
| PsycINFO | Knowledge management/ or knowledge transfer, (knowledge adj3 (translat* or exchang* or transfer* or diffusion or disseminat*)).tw., complex*.tw. , innovation, (organization* or organisation*).tw., (innovat* adj3 diffus*).tw. , health care delivery/ or health care reform |
| Scopus | (((TITLE-ABS-KEY(knowledge W/3 (translat* OR exchang* OR transfer* OR diffusion OR disseminat*))) AND (TITLE-ABS-KEY(complex*))) AND ((TITLE-ABS-KEY(("health care" OR healthcare) W/2 reform)) OR (TITLE-ABS-KEY(("health care" OR healthcare) W/2 (system or delivery))))) OR ((TITLE-ABS-KEY(innovat* W/3 diffus*)) AND ((TITLE-ABS-KEY(knowledge W/3 (translat* OR exchang* OR transfer* OR diffusion OR disseminat*))) AND (TITLE-ABS-KEY(complex*)))) OR (((TITLE-ABS-KEY(knowledge W/3 (translat* OR exchang* OR transfer* OR diffusion OR disseminat*))) AND (TITLE-ABS-KEY(complex*))) AND (TITLE-ABS-KEY(organization* OR organisation*))) AND ( LIMIT-TO(LANGUAGE,"English" ) ) |
| Web of Science | Knowledge NEAR/3 (translat* OR exchang* OR transfer* OR diffusion OR disseminat*)), TS=(organization* OR organisation*), TS=(complex*), TS=(innovat* NEAR/3 diffus*), TS=(("health care" OR healthcare) NEAR/2 (system or delivery)), TS=(("health care" OR healthcare) NEAR/2 reform).  Science Citation Index Expanded (SCI-EXPANDED) --1900-present  Social Sciences Citation Index (SSCI) --1956-present  Conference Proceedings Citation Index- Science (CPCI-S) --1996-present  Conference Proceedings Citation Index- Social Science & Humanities (CPCI-SSH) --1996-present |
| Authors search Medline, Cinhal, Google Scholar sites Joanna Briggs, McMaster University | Straus, S., Greenhalgh, T., Graham, I., Grimshaw, J., Berta, W., Kitson, A., Scott, S., Estabrooks, C., Logan, J., Rogers, E., Pettigrew, M., Pawson, R., Grol, R., Fineout-Overholt,, Raycroft-Malone, J |
